# Proinflammatory T cell polarization is already present in patients with early knee osteoarthritis

**DOI:** 10.1186/s13075-020-02410-w

**Published:** 2021-01-22

**Authors:** Nils Rosshirt, Richard Trauth, Hadrian Platzer, Elena Tripel, Timo A. Nees, Hanns-Martin Lorenz, Theresa Tretter, Babak Moradi

**Affiliations:** 1 Clinic for Orthopedic and Trauma Surgery, University Hospital Heidelberg, Schlierbacher Landstr. 200a, Heidelberg, 69118 Germany; 2 Department of Internal Medicine V, Division of Rheumatology, University Hospital Heidelberg, Heidelberg, Germany; 3 Clinic of Orthopedic and Trauma Surgery, University of Kiel, Arnold-Heller-Straße 3, Kiel, 24105 Germany

**Keywords:** Early OA, T cell polarization, T helper cells, Inflammatory T cells, Synovial membrane, Synovial fluid, Chemokine receptor, Cytokines

## Abstract

**Background:**

Investigating the pathophysiological mechanisms of early osteoarthritis (OA) is of utmost interest since this stage holds the strongest promise for therapeutic interventions. The aims of this study were to analyze if synovial inflammation is already present in early OA and to characterize the involved cell populations, by investigating synovial fluid (SF) and synovial membrane (SM) of early OA patients for the presence and polarization status of CD4 T cells.

**Methods:**

A quantitative analysis of CD4^+^ T cell infiltration in SF and SM compared to peripheral blood (PB) was performed in patients with early stages of OA. We further investigated intracellular staining (ICS), surface marker, and chemokine receptor expression profiles of CD4^+^ T cells in SF, SM, and PB, as well as cytokine expression in native SF and PB. Matched samples of SF, SM, and PB were harvested from 40 patients with early OA at the time of surgery. Early OA was confirmed by independent surgeons intraoperatively. Samples were analyzed by flow cytometry for surface markers and cytokines, which are preferentially expressed by distinct T cell subsets (Th1, Th2, Th17, regulatory T cells). Furthermore, we analyzed native SF and PB supernatants using MACSPlex for multiple cytokine expression profiles.

**Results:**

SF and SM showed a distinct infiltration of CD4^+^ T lymphocytes, with significantly increased expression of chemokine receptors CXCR3/CCR5, cytokine IFN-γ (preferentially expressed by Th1 cells), and CD161 (preferentially expressed by IL-17 producing Th17 cells) compared to PB. Furthermore, the percentage of CD4^+^ T cells polarized to Treg was significantly increased in SM compared to SF and PB. No significant differences were observed for CCR3 and CCR4 (preferentially expressed by Th2 cells), although IL-4 values were significantly higher in SM and SF compared to PB. Cytokine analysis showed comparable results between PB and SF, with only IL-6 being significantly increased in SF.

**Conclusions:**

Early OA joints show already significant inflammation through CD4^+^ T cell infiltration, with predominant Th1 cell polarization. Inflammation seems to be driven by direct proinflammatory cell interaction. Cytokine signaling seems to be negligible at the site of inflammation in early OA, with only IL-6 being significantly increased in SF compared to PB.

**Supplementary Information:**

The online version contains supplementary material available at 10.1186/s13075-020-02410-w.

## Background

Treatment of osteoarthritis (OA) continues to be challenging for patients, treatment providers, and health care systems worldwide. With only limited non-surgical options to treat OA symptoms and only surgical options for end-stage OA, it cannot be stated that we have reached a satisfying level. The impact of cellular and molecular pathways contributing to OA pathophysiology is being increasingly recognized to the same amount that the concept of wear and tear is questioned. Synovial inflammation has been identified as an independent factor significantly contributing to OA pathology. Modulation of inflammatory pathways has been successful in delaying OA progression in animal models [1–3]. Studies investigating the inflammatory cell infiltrate have identified T cells and macrophages as the main mononuclear cells (MNC) in the synovial fluid (SF) and membrane (SM). Flow cytometry and immunohistological studies revealed that CD4^+^ T helper cells present an activated phenotype and are polarized distinctly to proinflammatory subsets (Th1), overweighing the immunomodulatory regulatory T cells (Treg) in OA joints. Thus, Th1 infiltration is increasingly recognized as pivotal in OA pathology [4–8]. But these previous studies have mainly focused on joint-derived samples of patients with advanced OA. In contrast, early OA has widely been ignored so far [7–9]. Since end-stage OA is the result of chronic progressive disease with outbalanced joint-homeostasis developing over decades, it might be hard to decipher the impact of these inflammatory patterns on OA pathogenesis if we continue to analyze only final disease stages. Thus, it is pivotal to investigate the cellular and molecular inflammatory processes in early OA joints. Knowledge of the initial inflammatory processes in early OA might prove essential for establishing a new treatment window specifically in view of the limited regenerative capability of hyaline cartilage and the irreversible chronic changes that occur in the whole joint during OA progression. Assessment of early OA is indispensable in the search for biomarkers as a diagnostic tool. OA pathology has a temporal pattern, and cartilage, bone, and synovial matrix biomarkers show a positive association with the progression of knee OA [10]. Changes in cartilage and synovial fluid metabolism occur before any quantifiable structural damage can be recognized [11]. Although several publications suggest markers of early OA, an established diagnostic tool is still missing [12–14]. This is to our best knowledge the first study to perform flow cytometry analysis of tissue samples from early OA patients that represent a clearly defined group as categorized by independent surgeons intraoperatively. Furthermore, analysis of matched SF, SM, and peripheral blood (PB) samples from early OA patients is still missing.

## Methods

### Study population

A total of 40 patients with arthroscopic or MRI findings of early osteoarthritis of the knee (29 women, 11 men) were enrolled in this study. All patients included had a Kellgren-Lawrence grade 0–I, as confirmed by two-plane radiographs, and were scheduled for arthroscopic surgery at our hospital. No patient included underwent surgery directly after trauma. The minimum time after trauma/onset of symptoms was 6 weeks, and the median was 8 months (Table 1). International Cartilage Repair Society (ICRS) grade was assessed by independent surgeons intraoperatively. All patients included had ICRS grades I–IV in at least two compartments (5%) or grades II–IV in one compartment (95%) and met the criteria of early OA as previously suggested by Luyten et al. [15, 16]. None of the patients had a history of underlying inflammatory disease, intake of DMARD, intra-articular injection of corticosteroids and hyaluronic acid, or regular intake of NSAID. Systemic inflammatory parameters (CRP, WBC) were within the physiological range at the time of surgery, except for one patient (CRP 13.4 mg/dl). The mean age of the study population was 41.7 ± 14.3 years. Underlying pathologies for arthroscopic knee surgery were meniscal tear (40%), traumatic anterior cruciate ligament (ACL) tear (5%), and localized cartilage damage (55%) (Table 1). Subgroups were comparable concerning clinical data. Due to the methodological setup, e.g., limitations concerning cell number, not all patients could be included in every subgroup analysis. The local ethics committee approved this study (S-333/2007). Written informed consents from all patients were obtained prior to study enrolment.

**Table 1:** Characteristics of the study population

	Total study population	Surface marker/MACSPlex	ICS
Number of patients, *n*	40	31	9
Gender, *n* (%)			
Male	11 (27.5%)	10 (32.2%)	1 (11.1%)
Female	29 (72.5%)	21 (67.8%)	8 (88.9%)
Age at surgery, years	41.7 ± 14.3	41.3 ± 14.1	43 ± 14.9
Mean ± SD (IQR)	(28.4–51.2)	(28–52.5)	(30–47)
Operation side, *n* (%)			
Right	17 (42.5%)	14 (45.2%)	3 (33.3%)
Left	23 (57.5%)	17 (54.8%)	6 (66.6%)
BMI, kg/m^2^	27.9 ± 6.4	28.5 ± 7.2	25.7 ± 4.4
Mean ± SD (IQR)	(23.2–30.3)	(23.9–31.4)	(22.6–29.4)
Leucocytes/nl	7.01 ± 1.9	7.3 ± 1.9	6.38 ± 1.8
Mean ± SD (IQR)	(5.9–7.9)	(5.9–7.94)	(5.8–7.9)
C-reactive protein, mg/dl	3.3 ± 1.9	3.2 ± 2.2	3.4 ± 1.1
Mean ± SD (IQR)	(2–4)	(2.0–3.85)	(2–4)
Time from onset of symptoms/trauma to operation in months, *n* (%)			
1–3	2 (5%)	2 (6.4%)	0
4–6	16 (40%)	13 (42%)	3 (33.3%)
7–9	5 (12.5%)	4 (12.9%)	1 (11.1%)
10–12	7 (17.5%)	4 (12.9%)	3 (33.3%)
> 12	10 (25%)	8 (25.8%)	2 (22.2%)
ICRS grade			
I–IV in ≥ 2 compartments	2 (5%)	2 (6.5%)	0
II–IV in 1 compartment	38 (95%)	29 (93.5%)	9 (100%)
Pathology, *n* (%)			
Meniscus tear	16 (40%)	14/31 (45.1%)	2/9 (22.2%)
ACL tear	2 (5%)	2/31 (6.5%)	0/9
Localized cartilage damage	22 (55%)	15/31 (48.4%)	7/9 (77.7%)

Demographic and clinical parameters of the study population are shown. Values are shown as mean ± standard deviation (range) or as number (p%). ICRS grade was evaluated by independent surgeons intraoperatively

*IQR* interquartile range, *BMI* body mass index, *K & L score* Kellgren and Lawrence score (radiological score), *ICRS grade* International Cartilage Repair Society grade (arthroscopical grading), *ACL* anterior cruciate ligament

### Sample collection

The synovial fluid (SF), synovial membrane (SM), and peripheral blood (PB) were harvested at the time of surgery. SF was aspirated prior to the establishment of the arthroscopic portals into sterile tubes and further processed as described below. SM biopsies were performed intra-operatively from the suprapatellar pouch as previously described [7]. Heparinized PB samples were taken concurrently at the time of surgery.

### Sample preparation

Two hundred microliters of native SF prior to further processing and 200 μl of PB supernatants after density gradient centrifugation (see below) were harvested for MACSPlex analysis (Miltenyi Biotec, Germany) and deep-frozen at − 80 °C with a maximum time between sample collection and cryopreservation of 30 min. The remaining volume of SF samples was treated with bovine testicular hyaluronidase (10 mg/ml, Sigma-Aldrich, USA) for 30 min at 37 °C and washed twice with PBS. SM samples were rinsed twice with phosphate-buffered saline (PBS), minced finely with sterilized scissors, and digested with collagenase B (1 mg/ml; Roche, USA) and bovine testicular hyaluronidase IV (2 mg/ml; Sigma-Aldrich, USA) at 37 °C for 2 h in RPMI 1640 culture medium (Invitrogen, USA) supplemented with 10 μg/ml penicillin-streptomycin (Invitrogen, USA) and 5% FCS (Biochrom AG, Germany). The cell suspension was filtered through a 100-μm (BD Biosciences, USA) and a 40-μm pore-size cell strainer (EMD Millipore, USA) to remove any undigested tissue. The filtered cell suspension was washed twice with PBS. Mononuclear cells were isolated from heparin anti-coagulated whole blood, SF, and SM cell suspensions using Ficoll-PaqueTM PLUS (GE Healthcare, USA) density gradient centrifugation. T cells were isolated from PB, SF, and SM mononuclear cells by CD3 MACS bead separation (Miltenyi Biotec, Germany).

### Flow cytometry analysis of cell surface markers and intracellular staining

Multicolor flow cytometry was used to identify CD3^+^CD4^+^ T cell subsets by their preferential expression of surface and intracellular markers. In brief, for the staining of regulatory T cell (Treg) and T helper (Th1, Th2, Th17) cell surface markers, CD3^+^ MACS-isolated T cells from PB, SF, and SM were washed twice in staining buffer, blocked with FcR blocking reagent (Miltenyi Biotec, Germany), and then stained for 30 min at 4 °C with the following monoclonal antibodies (mAb) in two setups: VioBlue-labeled mAb against CD4 (clone VIT4, Miltenyi Biotec, Germany), Alexa Fluor 488-labeled mAb against CXCR3 (clone 1C6), PE-labeled mAb against CCR4 (clone 1G1), PE-Cy7-labeled mAB against CD25 (clone 2A3), Alexa Fluor 647-labeled mAb against CCR3 (clone 2A3), and APC-Cy7 labeled mAb against CCR5 (clone 2D7) in one setup; E-Fluor 450-labeled mAb against CD127 (clone eBioRDR5, E-Bioscience, USA), FITC-labeled mAb against CD161 (clone DX12), PE-Cy7-labeled mAb against CCR6 (clone 11A9), APC-labeled mAb against CD25 (clone 2A3), PE-labeled mAb against CCR4 (clone 1G1), and APC-Cy7-labeled mAb against CD4 (clone RPA-T4) in another setup. Before flow cytometric detection, 0.5 μg/ml 7-AAD (eBioscience; USA) was added to the cell suspensions, to exclude cell debris and dead cells.

For the staining of intracellular markers, MACS CD3-isolated T cells from PB, SF, and SM were taken into culture at a final density of 10^6^ T cells/ml and incubated for 10 h at 37 °C/5% CO_2_. Cell cultures were stimulated with phorbol-myristate-acetate (PMA) (50 ng/ml) and ionomycin (1 μg/ml). After 4 h of activation, brefeldin A (5 μg/ml, Sigma-Aldrich, Germany) was added for another 6 h. After a total of 10 h of activation, T cells were collected, washed twice in FACS buffer, and blocked with FCS blocking reagent. For surface marker expression, PE-Cy7-conjugated mAb against CD3 (clone SK7), APC-Cy7-conjugated mAb against CD4 (clone RPA-T4), and VioBlue-labeled mAb against CD8 (clone BW135/80, Miltenyi Biotec, Germany) were used, and staining was performed at 4 °C for 30 min. Following surface staining, T cells were fixed and permeabilized using cytofix/cytoperm reagent and then stained with APC labeled anti-IFN-γ (clone B27), FITC-labeled anti-IL-4 (clone MP4-25D2), and PE-labeled anti-IL-17A (clone N49-653). Cytokine values for CD3^+^CD4^+^CD8^−^ T cells were measured by flow cytometry.

Flow analysis was performed using a MACSQuant Analyzer (Miltenyi Biotec, Germany), which is a 7-channel flow cytometer. The total number of surface markers analyzed exceeded the channel number. Therefore, two antibody panels were needed. Data analysis was performed using FlowJo 9.6 (Treestar, USA). The cutoff for all cell surface markers was defined based on fluorescence minus one (FMO) controls. Antibodies and cell preparation solutions were purchased from BD Biosciences, USA, if not stated otherwise.

### Cytokine analysis

Native SF and PB sera were analyzed by the MACSPlex 12 Kit (Miltenyi Biotec, Germany). All working steps were carried out according to the manufacturer’s instructions, and washing procedures were performed with a centrifuge. Flow analysis was performed using MACSQuant Analyzer (Miltenyi Biotec, Germany). Data analysis was performed using Flow Logic™ (V7.2.1) and BeadLogic™ (V7) (Miltenyi Biotec, Germany).

### Statistical analysis

Data are presented as arithmetic median (IQR), if not stated otherwise. Gaussian distribution was assessed by the Kolmogorov-Smirnov test. To compare concurrent SF, SM, and PB samples, we used the Friedman test for non-parametric data with Wilcoxon signed-rank post hoc test and Bonferroni correction. Wilcoxon signed-rank test with Bonferroni correction was used for MACSPlex analysis. *p* values < 0.05 were considered significant. Statistical analysis was performed using SPSS 22.0 (IBM SPSS Inc., USA).

## Results

### Presence of T cells in OA joints and peripheral blood

Frequencies of MACS-isolated T lymphocytes of joint-derived samples (SF, SM) were analyzed by flow cytometry and compared to concurrent PB samples. PB revealed higher cell numbers with a mean of 39.9 × 10^5^ ± 6 × 10^5^ T cells compared to 0.96 × 10^5^ ± 0.3 × 10^5^ (SF) and 0.08 × 10^5^ ± 0.02 × 10^5^ (SM), as expected (Table 2). To further estimate the T cell percentage in SF and SM samples, the relation between T cell numbers and acquired sample volume or sample weight was calculated. Although still lower than PB (558.1 ± 88.5 cells/μl), SF and SM showed a distinct infiltration with a mean of 51.3 ± 21.3 T lymphocytes per microliter (SF) and 42.5 ± 12.7 T lymphocytes per milligram (SM), respectively. SF and SM contained comparable proportions of T lymphocytes that stained positive for CD4 (38.8% in SF and 44.3% in SM), albeit significantly lower than PB (71.2%).

**Table 2:** CD4^+^ T cell infiltration in the peripheral blood (PB) and joint-derived samples (SF, SM)

	PB	SF	SM	*p* values		
				PB:SF	PB:SM	SF:SM
*n*	18	18	18			
Sample volume/weight (PB, SF: ml; SM: g)	7.6 ± 0.4 (6.6–8.5)	4.2 ± 1.1 (1.8–6.5)	0.22 ± 0.03 (0.15–0.28)	n.a.	n.a.	n.a.
CD3^+^ MACS-isolated T lymphocytes of total living cells	92.2 (88.2–93.8)	46.8 (32.0–62.3)	12.3 (4.9–21.3)	0.0002***	0.0002***	0.0004***
Cell count of CD3^+^ MACS-isolated T lymphocytes	39.9 × 10^5^ ± 6 × 10^5^ (26 × 10^5^–53 × 10^5^)	0.96 × 10^5^ ± 0.3 × 10^5^ (0.25 × 10^5^–1.6 × 10^5^)	0.08 × 10^5^ ± 0.02 × 10^5^ (0.03 × 10^5^–1.3 × 10^5^)	n.a.	n.a.	n.a.
CD4^+^ T cells % of T lymphocytes	71.2 (60.6–81.1)	38.8 (27.5–49.4)	44.3 (36.5–49.4)	0.0002***	0.0002***	0.472
Cell count of T lymphocytes per sample volume/weight (PB/SF: cells/μl; SM: cells/mg	558.1 ± 88.5 (369.4–746.8)	51.3 ± 21.3 (7–95.5)	42.5 ± 12.7 (16.2–68.8)	n.a.	n.a.	n.a.

Number and mean volume/weight ± standard deviation (CI) of acquired and analyzed samples are given. Further, percentage rate and mean cell count ± standard deviation (CI) of CD3^+^ MACS-isolated T lymphocytes and the percentage of CD4^+^ MACS-isolated T lymphocytes in the peripheral blood (PB), synovial fluid (SF), and synovial membrane (SM) of early OA patients are shown. The cell count was taken in relation to the acquired sample volume/weight to illustrate the amount of inflammatory T cells in the different tissues. In brief, mononuclear cells were isolated from PB, SF, and SM by density gradient centrifugation. Further, T lymphocytes were isolated by CD3^+^ magnetic cell separation (MACS), stained with monoclonal antibodies (mAb) against CD4, and analyzed by flow cytometry. Data is shown as median (IQR), if not stated otherwise. Significant differences are marked with asterisks: *p < 0.05; **p < 0.01; ***p < 0.001

*n.a.* not applicable, *MACS* magnetic activated cell sorting, *IQR* interquartile range

### Comparison of T cell surface marker and chemokine receptor status in OA joints and peripheral blood

To evaluate T cell surface marker expression quantitatively, we isolated CD4^+^ T cells from PB, SF, and SM and used flow cytometry for detection of CXCR3, CCR5 (preferentially expressed by Th1 cells), CCR3, CCR4 (preferentially expressed by Th2 cells), CCR6, and CD161 (preferentially expressed by Th17 cells) as well as Treg-specific CD25 and CD127. All analyzed surface markers (chemokine and cytokine receptors) could be detected on synovial T cell surfaces. Table 3 presents the median (IQR) for the surface markers investigated in this study.

**Table 3:** Comparison of CD4^+^ T cell surface marker/chemokine receptor status in the peripheral blood (PB) and joint-derived samples (SF, SM)

CD4^+^ T cell subset		PB	SF	SM	*p* values		
					PB:SF	PB:SM	SF:SM
Th1	***n***	9	9	9			
	**CD4**^**+**^ **T cells**						
	***CXCR3***	10.0 (8.3–22.4)	66.2 (62.6–84.2)	76.9 (58.0–96.0)	0.008**	0.008**	0.515
	***CCR5***	13.4 (6.4–17.7)	76.1 (73.6–89.8)	79.9 (74.0–93.5)	0.008**	0.008**	0.594
Th2	***CCR3***	29.5 (22.0–32.0)	33.9 (20.3–52.8)	57.9 (26.5–85.8)	0.173	0.051	0.110
	***CCR4***	45.2 (36.6–56.6)	22.8 (8.9–37.3)	61.4 (24.1–87.7)	0.139	0.314	0.021*
Th17	***CCR6***	32.6 (25.7–37.1)	31.7 (28.7–44.3)	47.2 (18.8–81.3)	0.859	0.173	0.594
	***CD161***	4.6 (4.0–8.7)	24.4 (17.1–34.8)	32.7 (21.9–42.1)	0.011*	0.008**	0.678
Treg	**(*CD25***^***+/high***^***CD127***^***low/−***^**)**	3.6 (2.8–5.1)	6.3 (2.7–9.2)	12.3 (9.7–21.6)	0.139	0.008**	0.066

Sample number and percentage rates of CD4^+^ T cells stained positive for CXCR3, CCR5 (*preferentially expressed by Th1 cells*), CCR3, CCR4 (*preferentially expressed by Th2 cells*), CCR6, CD161 (*preferentially expressed by Th17 cells*), and CD25^+/high^CD127^low/−^ (*Treg*) are shown for the peripheral blood (PB), synovial fluid (SF), and synovial membrane (SM) of patients with early knee OA. In brief, mononuclear cells were isolated from PB, SF, and SM by density gradient centrifugation. CD3^+^ MACS-isolated T cells from PB, SF, and SM were stained with monoclonal antibodies (mAb) for the following surface markers: CD4, CD25, CD127, CXCR3, CCR5, CCR3, CCR4, CD161, and CCR6. Before flow cytometric detection, 7-AAD was added to the cell suspensions to exclude cell debris and dead cells. The cutoff for all cell surface markers was defined based on fluorescence minus one (FMO) controls. Data is shown as median (IQR). Significant differences are marked with asterisks: *p < 0.05; **p < 0.01; ***p < 0.001

Synovial T cells in SF and SM showed a predominant and in comparison with PB significantly increased expression of CXCR3 (*p* < 0.01), CCR5 (*p* < 0.01), and CD161 (*p* < 0.01 [SM] and *p* < 0.05 [SF]). No significant differences were found between SF and SM for these markers. CCR4 showed significantly higher expression rates on T cells isolated from SM, compared to SF (*p* = 0.021). No significant differences were shown for chemokine receptors CCR3 and CCR6 between the groups. CD4^+^CD25^+/high^CD127^low/−^ regulatory T cells were significantly enriched in SM compared to PB (*p* < 0.01).

**Fig. 1:**
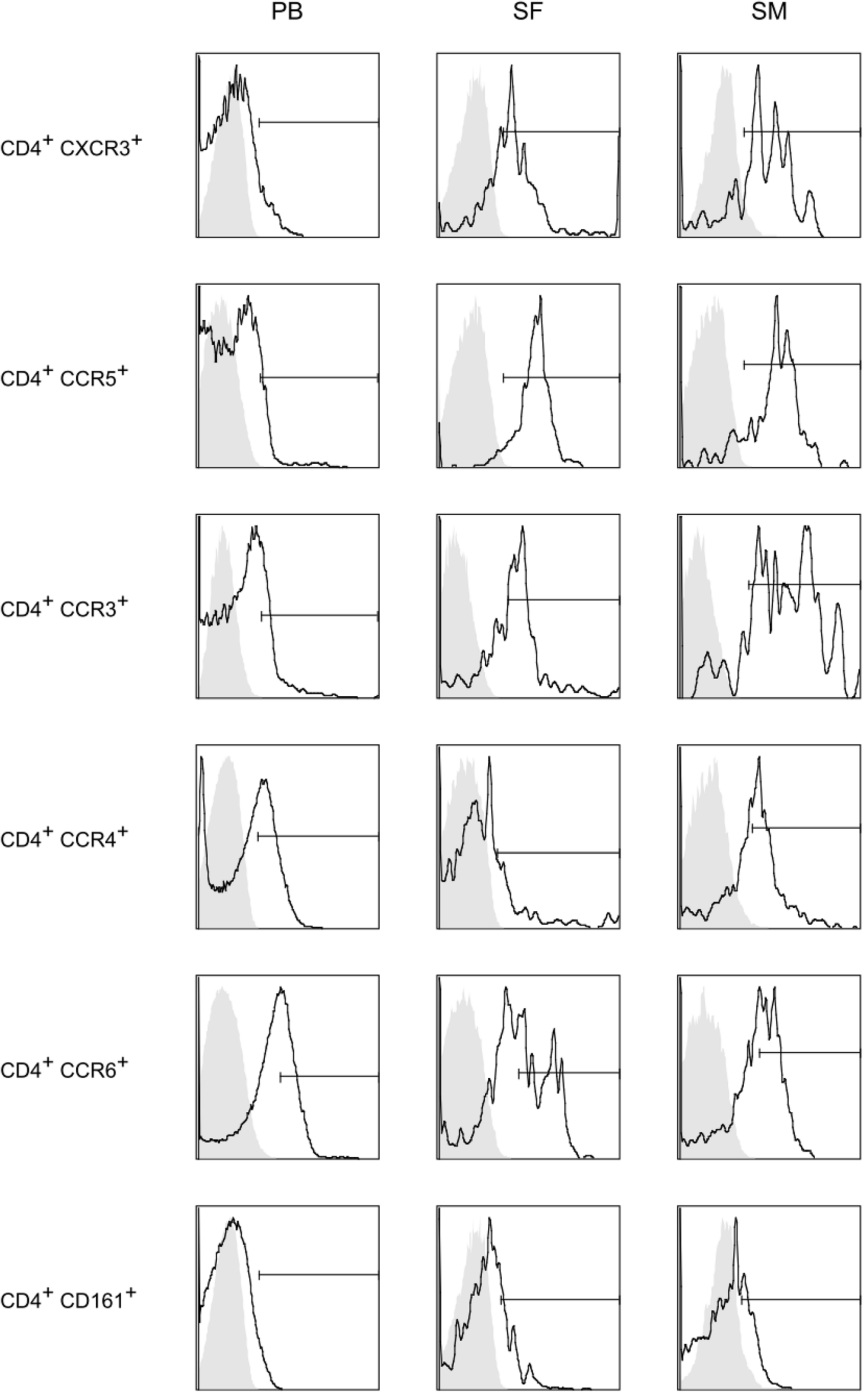
Flow cytometry analysis of CD4^+^ T cell surface marker expression. Representative flow cytometry histograms of CD4^+^ T cell surface marker expression are shown. In short, mononuclear cells were isolated from PB, SF, and SM by density gradient centrifugation. CD3^+^ MACS-isolated T cells from PB, SF, and SM were stained with monoclonal antibodies (mAb) for the following surface markers: CD4, CD25, CD127, CXCR3, CCR5, CCR3, CCR4, CD161, and CCR6. Before flow cytometric detection, 7-AAD was added to the cell suspensions to exclude cell debris and dead cells. The cutoff for all cell surface markers was defined based on fluorescence minus one (FMO) controls (shown as gray overlay population). mAb, monoclonal antibody; MACS, magnetic-activated cell sorting

Furthermore, analysis of CD4^+^ T cells co-expressing CXCR3 and CCR5 showed that the majority of CD4^+^CXCR3^+^ T cells in SF and SM stained also positive for CCR5 and vice versa (55.4% [SF] and 67.1% [SM]), whereas only 2.6 out of 10.0% of CD4^+^CXCR3^+^ and CD4^+^CCR5^+^ T cells showed that expression pattern in PB. In contrast to PB, our data revealed highly significant polarization towards CD4^+^CXCR3^+^CCR5^+^ T cells with 55.4% (SF) and 67.2% (SM), compared to 2.6% in PB (*p* < 0.01). Co-expression of CCR3 and CCR4 was significantly higher in SM with 48.4% compared to SF (12.0%; *p* = 0.038). No significant differences were found, when compared to PB (14.9%; *p* = 0.051). Also, analyzing the co-expression of CD161 and CCR6, SM (16.8%) and SF (12.2%) had significantly higher values than PB (3.2%) (Fig. 1, Table 4).

**Table 4:** Co-expression of CD4^+^ T cell surface marker/chemokine receptors in the peripheral blood (PB) and joint-derived samples (SF, SM)

	PB	SF	SM	*p* values		
				PB:SF	PB:SM	SF:SM
*n*	9	9	9			
CD4^+^ T cells						
*CXCR3*^*+*^*/CCR5*^*+*^	2.6 (0.9–3.1)	55.4 (53.1–74.9)	67.2 (46.9–88.9)	0.003**	0.008**	0.678
*CCR3*^*−*^*/CCR4*^*−*^	0.6 (0.3–1.0)	31.5 (16–40.1)	5.8 (1.1–18.0)	0.008**	0.021*	0.051
*CCR3*^*−*^	1.0 (0.5–2.1)	33.8 (18.2–50.9)	12.4 (6.1–27.2)	0.008**	0.008**	0.066
*CCR4*^*−*^	1.1 (0.4–1.3)	47.9 (27.8–52.2)	12.6 (3.8–28.6)	0.008**	0.011*	0.021*
*CCR3*^*+*^*/CCR4*^*+*^	14.9 (7.7–17.6)	12.0 (4.9–15.5)	48.4 (8.5–78.2)	0.593	0.051	0.038*
*CXCR3*^*−*^*/CCR5*^*−*^	11.2 (6.0–13.0)	0.14 (0.1–1.0)	0.08 (0–0.6)	0.008**	0.008**	0.594
*CCR5*^*−*^	14.3 (6.8–15.4)	1.2 (0.3–2.5)	2.0 (1.1–5.3)	0.008**	0.011*	0.260
*CXCR3*^*−*^	11.8 (6.2–14.5)	1.4 (0.6–4.2)	2.1 (0–3.1)	0.028*	0.011*	0.767
*CD161*^*+*^*/CCR6*^*+*^	3.2 (2.4–6.1)	12.2 (5.3–14.7)	16.8 (5.0–34.2)	0.003**	0.021*	0.594
*CCR4*^*−*^	1.2 (0.9–8.7)	5.1 (3.0–8.7)	2.2 (0.7–3.7)	0.011*	0.314	0.139

Sample number and percentage rates of CD4^+^ T cells stained double-positive for CXCR3 and CCR5 (*preferentially expressed by Th1 cells*), CCR3 and CCR4 (*preferentially expressed by Th2 cells*), and CCR6 and CD161 (*preferentially expressed by Th17 cells*) are shown for the peripheral blood (PB), synovial fluid (SF), and synovial membrane (SM) of patients with early knee OA. Further, percentage rates of double-positive CD4^+^ T cells (CD4^+^CXCR3^+^CCR5^+^ Th1, CD4^+^CCR3^+^CCR4^+^ Th2, CD4^+^CD161^+^CCR6^+^ Th17) being also negative for the surface markers of the respective other T cell subtype are listed. Data is shown as median (IQR). Significant differences are marked with asterisks: *p < 0.05; **p < 0.01; ***p < 0.001

**Fig. 2:**
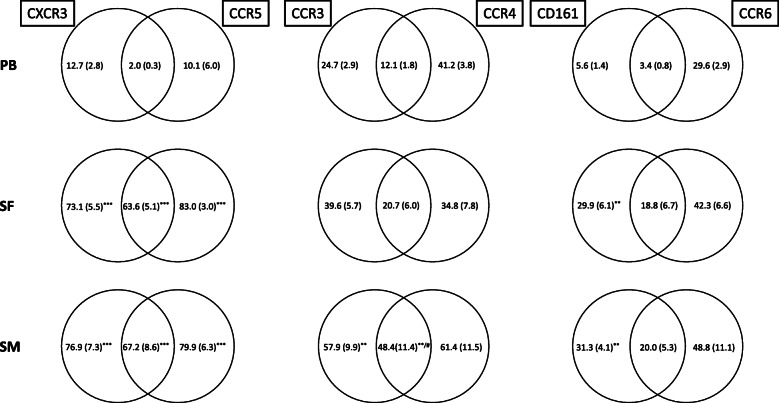
Co-expression of CXCR3/CCR5, CCR3/CCR4, and CD161/CCR6 on CD4^+^ T lymphocytes. Percentage rates of CXCR3/CCR5, CCR3/CCR4, and CD161/CCR6 co-expression on CD4^+^ T lymphocytes in the peripheral blood (PB), synovial fluid (SF), and synovial membrane (SM) of patients with early knee OA are shown. Surface marker expression was analyzed by flow cytometry, and the results are presented as a percentage of total CD4^+^ T cells. Data is shown as mean (SEM) (*n =* 12 paired samples). Significant differences between SF/PB and SM/PB are marked with asterisks (*); significant differences between SF/SM are marked with a hash (^**#**^): *p < 0.05; **p < 0.01; ***p < 0.001; ^**#**^*p* < 0.05

To further evaluate the expression pattern of the mentioned chemokine receptors and surface markers on CD4^+^ T cells, we analyzed how many double-positive CD4^+^CXCR3^+^CCR5^+^-stained T cells did not express CCR3/CCR4 and vice versa. Interestingly, in SM and SF as well as in PB, a big proportion of CD4^+^CXCR3^+^CCR5^+^ T cells stained also positive for CCR3 and CCR4. Whereas in PB 0.6% out of 2.6% (23.1%) of CD4^+^CXCR3^+^CCR5^+^ T cells stained negative for CCR3/CCR4, SF showed a higher percentage with 31.5% out of 55.4% (56.7%) and SM showed a lower fraction of 5.8% out of 67.2% (8.6%). Similar results were obtained for CD4^+^CD161^+^CCR6^+^ T cells with 1.2% out of 3.2% (37.5%) staining negative for CCR4 in PB, whereas 5.1% out of 12.2% (41.8%) in SF and 2.2 out of 16.8% (13.1%) in SM showed no co-expression of CCR4 (Table 4, (Fig. 2).

CD4^+^CCR3^+^CCR4^+^ and CD4^+^CD161^+^CCR6^+^ T cells presented a clear difference between peripheral and synovial T cells regarding the exclusive expression of chemokine receptors. While 11.2 out of 14.9% of CD4^+^CCR3^+^CCR4^+^ T cells in PB did not co-express CXCR3 and CCR5, very few T cells showed this exclusive expression pattern in SM and SF.

### Cytokine secretion of activated CD3^+^CD4^+^CD8^−^ T cells in OA joints and peripheral blood

We further examined the polarization of the T cell population by utilizing flow detection of Th cell-specific intracellular markers to define the following T helper cell subsets: Th1 (IFN-γ), Th2 (IL-4), and Th17 (IL-17A), and compared synovial samples to concurrent samples of PB (Table 5). Whereas in PB only a small proportion of T cells stained positive for T helper cell subsets, synovial T cells isolated from SF and SM showed a significantly increased polarization towards Th1 cells with 55.8% in SF and 42.8% in SM compared to 14.2% in PB and Th2 cells with 2.9% in SF and 2.0% in SM compared to 0.6% in PB. No significant differences were detected for Th17. Interestingly, polarization towards Th2 and Th17 was much lower, than expected from single surface marker analysis.

**Table 5:** Cytokine secretion of activated CD3^+^CD4^+^CD8^−^ T cells in the peripheral blood (PB) and joint-derived samples (SF, SM)

	PB	SF	SM	*p* values		
				PB:SF	PB:SM	SF:SM
*n*	9	9	9			
Th1 (*IFN-γ*)	14.2 (12.3–24.6)	55.8 (40.1–85.6)	42.8 (19.0–53.8)	0.008**	0.038*	0.051
Th2 *(IL-4*)	0.6 (0.5–1.9)	2.9 (1.1–4.0)	2.0 (1.6–4.7)	0.008**	0.008**	0.953
Th17 (*IL-17A*)	0.9 (0.7–1.5)	1.6 (0.8–2.2)	2.0 (1.2–2.2)	0.678	0.086	0.678

Percentage rates of CD3^+^CD4^+^CD8^−^ T cells stained positive for IFN-γ (Th1), IL-4 (Th2), and IL-17A (Th17) are shown for the peripheral blood (PB), synovial fluid (SF), and synovial membrane (SM) of patients with early OA. Intracellular markers were stained with APC-labeled anti-IFN-γ (clone B27), FITC-labeled anti-IL-4 (clone MP4-2502), and PE-labeled anti-IL-17A (clone N49-653). Data is shown as median (IQR). Significant differences are marked with asterisks: *p < 0.05; ** *p* < 0.01

### MACSPlex analysis of cytokine expression pattern of native peripheral blood and synovial fluid supernatants

Matched samples of native SF and PB supernatants were analyzed for cytokine expression, using MACSPlex (Miltenyi, Germany).

Overall, the analyzed set of cytokines showed comparable results between PB and SF (Table 6). Significantly higher values were only shown for IL-6 in SF (19.45 ng/ml) compared to PB (1.18 ng/ml).

**Table 6:** MACSPlex analysis of cytokine concentration in supernatants of the peripheral blood (PB) and native synovial fluid (SF)

	PB	SF	*p* values
*n*	10	10	
GM-CSF	0.61 (0.45–1.02)	0.88 (0.40–2.04)	0.655
IFN-γ	18.98 (10.53–33.80)	15.49 (9.38–30.26)	0.715
IL-2	5.2 (2.74–7.69)	5.51 (3.64–7.96)	0.655
IL-4	102.67 (67.65–146.47)	87.37 (20.43–124.88)	0.138
IL-5	2.21 (1.21–4.16)	2.81 (1.32–5.34)	0.715
IL-6	1.18 (0.62–1.84)	19.45 (2.77–71.54)	0.013*
IL-9	46.0 (40.72–54.29)	40.81 (39.57–63.70)	1.000
IL-10	10.79 (8.86–19.85)	8.12 (5.01–15.16)	0.953
IL-12	9.54 (6.05–21.58)	6.96 (4.25–10.51)	0.093
IL-17A	3.34 (1.79–4.02)	3.84 (1.29–4.81)	1.000
TNF-α	5.64 (4.43–7.72)	7.73 (4.20–198.95)	0.249

To evaluate multiple soluble immunomodulating cytokines in PB supernatants and native SF of early OA patients, we utilized the MACSPlex 12 Human Kit (Miltenyi, Germany). All work steps were performed according to the manufacturer’s instruction. Data is shown as median (IQR). Cytokine concentrations are given in pg/ml. Significant differences are marked with an asterisk: *p < 0.05

## Discussion

OA is a multifactorial disease affecting the whole joint. Reflecting recent literature, the model of sole mechanical “wear and tear” causing cartilage degradation and thus OA progression is obsolete. Overwhelming evidence ascribes inflammatory processes a pivotal role in the generation of OA symptoms as well as OA onset and progression. Nevertheless, expertise about T cell polarization and molecular mechanisms during the onset of the disease is limited. To our best knowledge, there is no study utilizing flow cytometry to analyze T cell subtypes in SF, SM, and PB of a clearly defined early OA group.

This study analyzed the expression of a broad variety of cytokines, chemokines, and other surface markers on CD4^+^ T cells in paired samples of SF, SM, and PB of patients with clearly defined early OA as proposed by Luyten et al. [15, 16], as well as cytokine expression in native SF compared to PB. Although staining of CD4 showed significantly lower percentages in SM and SF compared to PB, our results reveal a significantly increased expression of CCR5, CXCR3, and CD161 among joint-derived CD4^+^ T cells (SM and SF) compared to PB in early OA. The expression of CCR5 and CXCR3 were previously identified as biomarkers correlating with OA-related knee pain and OA severity in a group of end-stage OA patients, respectively [17].

Korchi et al. found signs of synovial inflammation and hypervascularization already 3 months after OA onset in an animal model of early OA by analyzing radiological and histological changes [18]. Interestingly, the amount of CD4^+^ T cells expressing CCR5 in SM and SF of our early OA patients is comparably high as previously described in SF of patients with different forms of rheumatic joint diseases such as RA, psoriatic arthritis, and ankylosing spondylitis [19, 20]. Nevertheless, one limitation of our study is that CD4 staining of MACS-isolated CD3^+^ T lymphocytes might be compounded by surface marker cleavage due to the performed enzymatic digestion, as previously described [21]. Other groups showed lower expression for CCR4 and CCR5 in end-stage OA compared to RA. CCR5 expression was also remarkably lower in these end-stage OA data than in our early OA group [22, 23]. Moreover, studies comparing the synovial membrane from early and late OA patients by histology and immunohistochemistry found a higher inflammatory potential in early OA compared to late OA [6, 24]. These findings indicate that inflammatory disease activity in OA joints is highest during the onset of the disease, possibly promoting cartilage degradation [25, 26]. Of course, the underlying pathology itself (ACL tear, meniscus injury, etc.) might induce inflammatory pathways to a certain extent, leading to a selection bias in our study. Several groups investigated inflammation in meniscal injury or ACL tear cohorts that might probably overlap with our study group. Synovial inflammation was found to be present in 43% of patients with meniscal injuries [27]. Interestingly, the report of Clair et al. also showed significantly increased synovial concentration of IL-6, but also MCP-1, MIP-1β, and MMP-3, which were not examined in our study [28]. Another group investigating the synovial inflammatory biomarkers 2 years after ACL tear showed no correlation to patient-reported outcomes after 5 years, concluding chronic inflammation is not mainly responsible for the clinical results [29]. However, obtaining tissue samples from early OA patients without a certain pathology is justifiably not possible due to ethical considerations.

Furthermore, our data presents an increased percentage of CD4^+^CD25^+/high^CD127^low/−^ Treg in SM compared to SF and PB, which is even higher than we previously published for end-stage OA [30]. This is highly interesting, and we hypothesize that these elevated Treg numbers in early OA are an attempt of the immune system to contain the ongoing immune response and redress balance between pro- and anti-inflammatory immune cells.

In general, conflicting results have been reported regarding cellular inflammation in end-stage OA. It seems that a number of different cells contribute to inflammatory processes. Some groups consider cells of the innate immune system to be of utmost importance in OA inflammation. Monocytes and macrophages were ascribed to have the highest impact on inflammation in end-stage OA [31]. Neutrophil granulocytes were shown to be the major source of cartilage-degrading enzymes in a murine model of early OA [32]. Other groups declared cells of the adaptive immune system, especially T cells to be in an activated status and highly involved in generating a proinflammatory milieu in OA [30, 33]. Possible reasons for these diverging results might be that specimens of end-stage OA patients are heterogenous regarding inflammatory activity and that experimental setups differ. In our study, we focused on T cells and T cell-specific surface markers as well as analyzing a broad spectrum of cytokines. It cannot be ruled out that cells of innate immunity interact with T cells and thereby influence synovial inflammation in early OA.

Besides, differing results regarding inflammation in early OA could be caused not only by a different experimental setup, but also by an accentuated bias. One limitation of our study is that due to ethical concerns and experimental setup, not all performed experiments were applicable to the whole study population. Above all, limited cell numbers isolated from SM samples were a bottleneck for some subanalyses.

Further, until today, the term “early OA” itself is not clearly defined and used for various and heterogenous joint pathologies. Patients with isolated cartilage lesions but otherwise healthy joints, as well as symptomatic patients with multiple joint surface defects who are at higher risk to develop manifest OA, were previously lumped together in several studies. The overall problem is that OA criteria, based on radiographic changes and clinical findings, have not been changed since 1986 [34], although it is commonly accepted that early OA (Kellgren and Lawrence 0–I) can rarely be diagnosed on plain radiographs. That is why we used anamnestic information, as well as arthroscopic assessment of knee joints by independent surgeons to ensure that the criteria of early OA, as proposed by recent publications, were fulfilled [15, 16].

Due to the vast plasticity of T cell surface marker expression, it is hard to define a distinct T cell-specific pattern, especially given that surface marker expression varies during migration and activation processes [35]. Nevertheless, in vitro data indicating a preferential expression pattern associated with T cell differentiation do exist: virtually, all IL-17-producing CD4^+^ T cells (Th17) were found to express CCR6 [36]. CXCR3 and CCR5 are preferentially expressed on activated Th1 cells [37, 38] and Th17 cells [39], but CXCR3^+^ and CCR5^+^ T cells were shown to produce the Th1-specific cytokine IFN-γ upon stimulation ex vivo [40, 41]. CCR4^+^ and CCR3^+^ T cells were shown to secrete Th2-specific IL-4 [40–42]. How stable these surface marker expression patterns actually remain in vivo is not known. To further validate our surface marker results, we performed ICS staining. This data confirmed significantly increased polarization to Th1 in SM and SF compared to PB, but also significantly increased detection of Th2-specific cytokine IL-4, albeit with a much lower percentage rate than expected (Table 5).

Chemokine receptors have a crucial role in leukocyte migration to sites of inflammation and thus regulation of immune responses. CCR5 is an essential chemokine mediating T cell recruitment to inflammatory sites, but also seems to play a role in the resolution of inflammation [43]. Diapedeses of T cells into inflamed SM or SF is thought to happen by selective migration via endothelial P- and E-selectin and CXCR3/CCR5-activating chemokines [20, 44, 45]. Besides these adhesion molecules, chemotactic cytokines CCL (CC chemokine ligand) 2, CCL3, and RANTES (regulated and normal T cell expressed and secreted) are thought to have the greatest impact on T cell infiltration into sites of inflammation. All of these have been detected in OA joints [23]. Furthermore, chemokine receptor expression is not a unique feature of T cells. Chemokines are abundantly present at inflammatory sites, also in chondrocytes and fibroblasts in the sublining synovial membrane and seem to perpetuate MMP release in the process of cartilage degradation [46]. In the study presented, we focused solely on chemokine expression of CD4^+^ T cells. Our data reveals a significant increase in the co-expression of CXCR3/CCR5 (CD4^+^CXCR3^+^CCR5^+^ T cell) in SM and SF compared to PB, which are preferentially expressed by (activated) Th1 cells. Hence, we hypothesize that this proinflammatory T cell subset plays the main role in early OA inflammation.

The MACSPlex results presented in our study showed a significantly elevated level only for IL-6 in SF compared to PB. No significant differences were found for the other tested cytokines. The heightened IL-6 level could be due to increased secretion of synovial fibroblasts as an effect of RANTES-induced stimulation [47]. Elevated serum levels of IL-6 were associated with knee cartilage loss in humans [48], and IL-6 levels were shown to be significantly increased in SF of end-stage OA compared to controls [49, 50]. Since anti-inflammatory drugs blocking the IL-6-receptor have been utilized in the second-line treatment of rheumatoid arthritis (RA) for more than a decade [51], it appears peculiar that research focusing on manipulation of IL-6 cascade in OA is scarce. One group demonstrated that IL-6 acts catabolic by inducing cartilage-degrading enzymes mainly via Stat3 (signal transducer and activator of transcription 3) signaling in a murine model of OA, and the administration of mAb against IL-6R was shown to alleviate osteoarthritic cartilage lesions [52]. Also, the application of tocilizumab (IL-6R blocker) intrathecally was shown to attenuate pain symptoms in a murine OA model, although the study setup is not transferable to humankind [53]. Further, several phytotherapeutics were shown to decrease IL-6 chondrocyte-secretion and thereby MMP13 expression [54, 55], postulating that this pathway is possibly a lever that could be used to develop new therapies, although with moderate validity.

Taken together, our data shows that cellular and humoral inflammation is present already in early OA. Assuming that these inflammatory patterns contribute to progressive joint damage by downstream activation of catabolic enzymes, as previously shown for end-stage OA [26], appears conclusive. However, to date, there is no therapeutic strategy aiming at inhibition of synovitis by modulating the molecular characteristics of proinflammatory Th1 cells.

## Conclusion

To our best knowledge, this is the first flow cytometry study implementing a complete set of SF, SM, and PB specimens of a clearly defined early OA group. It has to be stated that the literature focusing on patients with early OA is scarce, but even scarcer are studies comparing different stages of OA. Further studies comparing inflammatory parameters in early and late OA are warranted for a better understanding of the changes in inflammatory activity during disease progression. Only then can we achieve the possibility to discover new treatment strategies aimed at halting disease progression. Our data demonstrates that infiltration of inflammatory Th1 cells occurs already in early OA stages and in combination with the solitary increase of IL-6 ascribes direct cellular interaction in combination with humoral immunity and involvement in early OA pathogenesis. Therapeutic studies should focus on blocking IL-6 secretion and function, as well as modulation of Th1 cells to facilitate not only symptomatic, but also causal therapies for OA patients.

## Supplementary Information

**Additional file 1.** Antibody panel.

## Data Availability

The datasets used and analyzed during the current study are available from the corresponding author on reasonable request.

## Abbreviations

ACL: Anterior cruciate ligament
BMI: Body mass index
CCL: CC chemokine ligand
CCR: CC chemokine receptor
CXCR: CXC chemokine receptor
CD: Cluster of differentiation
CRP: C-reactive protein
DMARD: Disease-modifying anti-rheumatic drug
ICRS: International Cartilage Repair Society grade
IFN-γ: Interferon gamma
IL: Interleukin
IP-10: Interferon γ-induced protein 10 (CXCL10)
IQR: Interquartile range
K & L score: Kellgren and Lawrence score
mAb: Monoclonal antibody
MDC: Macrophage-derived chemokine (CCL22)
n.a.: Not applicable
NSAID: Non-steroidal anti-inflammatory drugs
OA: Osteoarthritis
PB: Peripheral blood
PDGF: Platelet-derived growth factor
RA: Rheumatoid arthritis
RANTES: Regulated on activation, normal T cell expressed and secreted (CCL5)
SF: Synovial fluid
SM: Synovial membrane
STAT3: Signal transducer and activator of transcription 3
Th: T helper cell
TNF-α: Tumor necrosis factor alpha
Treg: Regulatory T cells
